# Antioxidant Activity of *Moringa oleifera* and Olive *Olea europaea* L. Leaf Powders and Extracts on Quality and Oxidation Stability of Chicken Burgers

**DOI:** 10.3390/antiox11030496

**Published:** 2022-03-03

**Authors:** Marwa Ezz El-Din Ibrahim, Randah Miqbil Alqurashi, Fatimah Yousef Alfaraj

**Affiliations:** 1 Department of Food and Nutrition, College of Agriculture and Food Sciences, King Faisal University, Al-Ahsa 31982, Saudi Arabia; msoilman@kfu.edu.sa (M.E.E.-D.I.); fzmmafa@hotmail.com (F.Y.A.); 2 Department of Nutrition and Food Science, College of Home Economic, Helwan University, Cairo 11795, Egypt

**Keywords:** *Moringa oleifera* leaves, olive *Olea europaea* L. leaves, natural antioxidant, peroxide value, lipid oxidation, chicken burger, meat preservation

## Abstract

Oxidation is the main cause of quality deterioration in meat-based foods, such as burgers. Antioxidants inhibit the oxidation process; recently, natural antioxidants have gained interest, due to safety concerns. In this study, the effects of leaf powder and crude extracts of both Moringa oleifera and olive in chicken burgers were studied for their antioxidant potential in preventing fat oxidation during storage. Antioxidant activities were evaluated using DPPH (2,2-diphenyl-1-picrylhydrazyl). The results showed the highest DPPH radical scavenging with IC_50_ values of 2.397 ± 0.10 mg/mL in the Moringa leaf. Total phenolic content (TPC) was crude olive extract > crude Moringa extract > olive leaf > Moringa leaf. The total flavonoid content (TFC) was significantly higher in the olive leaf and its crude extract than in the Moringa leaf and its crude extract. The pH, total volatile nitrogen, and sensory properties were affected by the addition of olive and Moringa (leaf and crude extracts) to chicken burgers refrigerated for 20 days. The addition of Moringa and olive leaf powder decreased lipid oxidation and PV after 10 days of storage. In general, Moringa and olive leaf treatments slowed the deterioration of meat, suggesting their use as preservatives to extend the shelf-life of chicken burgers.

## 1. Introduction

The global production of white meat products, especially poultry meat, has increased because of its sensory (e.g., color, odor, flavor, and texture) attributes, and the consumer belief that white meat is healthier than red meat. One of the main goals of meat manufacturers is to provide consumers with fresh food in terms of color, flavor, and odor. However, white meat products are highly sensitive to spoilage and damage; for example, different methods of meat preparation, such as grinding, blending, and heating, cause fat oxidation [1,2,3]. Fat oxidation is the most important factor affecting the quality of meat. It can help develop pleasant aromas in some circumstances such as processing, handling, and storage [4]. Indeed, it is well recognized that chemicals formed from lipid oxidation play a key role in the production of the characteristic aromas associated with meat products, which are highly valued by consumers [5]. Lipid oxidation is a complex process which occurs in three different ways, each of which involves a series of complex reactions: autoxidation, enzymatic-catalyzed oxidation, and photo-oxidation. Autoxidation, which is a continuous free radical chain reaction, is the predominant process-causing lipid oxidation in meat [5]. Unsaturated fatty acids and oxygen interact mostly through autoxidation [6,7]. Oxygen must be activated, resulting in the formation of a singlet oxygen (^1^O_2_) or a reactive oxygen species, such as hydrogen peroxide (H_2_O_2_), a superoxide anion (O_2_^•−^), or a hydroxyl radical (OH•) [7]. Initiation occurs as hydrogen is abstracted from an unsaturated fatty acid. The resulting alkyl radical tends to be stabilized by double-bond rearrangement to form a conjugated diene or triene [8]. These alkyl radicals are the first free radicals that initiate lipid oxidation [9]. The alkyl radical produced during the initiation phase reacts with the molecular oxygen to form peroxy radicals (a radical coupling with an oxygen molecule). They are highly reactive and abstract hydrogen from adjacent lipids. A hydroperoxide and an alkyl radical are produced due to this reaction. The process is repeated when the new alkyl radical interacts with molecular oxygen to create new peroxy radicals [6]. Hydroperoxides and aldehydes, which are formed due to this reaction, are the most important breakdown products, and the main contributors to the volatile flavors in meat [10].

Oxidation leads to considerable changes in food properties, such as color, reduction in nutritional value [11], damage to the product, vitamin and unsaturated fatty acid depletion, generation of free radicals, development of unpleasant flavors, and shelf-life reduction [12]. Thus, oxidation results in the production of different compounds, which have adverse effects on the quality of meat and meat products [1,13]. This has led to a demand for techniques to increase the shelf-life, safety, and quality of poultry meat [14,15]. One of the methods used to address these issues involves the addition of antioxidants to food during manufacturing [16]. Butylhydroxytoluene (BHT) and butyl hydroxyanisole represent the most important chemical agents used to reduce fat oxidation; however, their use is only permitted within acceptable limits because they have toxic effects in addition to the odor imparted by phenols [17,18]. One important advantage of natural antioxidants is that they do not require safety tests before use. In addition, they may be more efficient than synthetic agents [19]. Therefore, the demand for natural antioxidants has increased in recent years. Consequently, customers are increasingly interested in using natural products instead of synthetic products [20]. Plants are the most important source of natural antioxidants because they contain bioactive compounds, such as flavonoids, carotenoids, tocopherols, and polyphenolic substances [21,22]. These contribute to the preservation and improvement of the quality of meat and meat products [1].

*Moringa oleifera* (Moringa) leaves and seeds are valuable sources of bioactive compounds [23]. Moringa is widely cultivated in Southeast Asia, mainly in Thailand, India, the Philippines, and Pakistan [24]. The compounds in Moringa, in addition to their important medicinal properties [23], function as effective antioxidants and inhibitors of bacterial and fungal growth. Moringa is rich in phenolic compounds that have been found to considerably inhibit oxidation in food [25,26]. The leaves of *Moringa oleifera* contain 11 phenolic acids (gallic acid, caffeic acid, chlorogenic acid, o-coumaric acid, p-coumaric acid, ellagic acid, gentisic acid, sinapic acid, and syringic acid) [27,28], flavonoids (primarily flavonol and glycoside: quercetin, rhamnetin, campferol, apigenin, and myricetin), and their derivatives (coumaroylquinic acids and their isomers, feruloylquinic, and caffeoylquinic) [29]. In addition, amino acids, minerals, vitamins, and beta-carotene are present in Moringa leaves and seeds [30,31].

Olive trees (*Olea europaea* L.), which are native to the Mediterranean Basin, have spread throughout the world and adapted to a variety of climatic conditions, despite the fact that the Mediterranean region is the most important in terms of olive production [32]. *O. europaea* is now found throughout Asia, America, and Oceania, owing mostly to the olive oil industry. Olive oil is a major component of the Mediterranean diet, is used in medicine, and is a source of lamp fuel. It now has wide applications in the modern cosmetic sector and in nutrition. However, regardless of the field in which it is used, the olive oil industry produces large amounts of waste, especially during the agricultural phase, which includes harvesting and oil production. Olive pulp and leaves are common by-products linked to pollution of soil and water [33]. The demand for olive leaves, as both whole and extract forms, have increased dramatically in the use of foodstuff to increase the value of the food and as a preservative of unsaturated fat-rich foods [34]. Phenolic compounds such as oleuropein, hydroxytyrosol, oleuropein, verbascoside, ligostroside, tyrosol, and tocopherol are present in large quantities in mature olive leaves [35,36,37]. These compounds contain groups of phenolic compounds that serve as antioxidants by chelating metals like copper and iron, which catalyze free radical production reactions, such as lipid oxidation [38]. In addition, olive leaves show antimicrobial activity against bacteria, fungi, and mycoplasma [39,40].

In recent years, the number of local and international consumers of chicken burgers has increased rapidly during the past ten years, and this food item has become highly preferred by consumers [2]. The substitute of red meat with chicken in the burger industry is gaining popularity because of their high-fat content and because there are no cultural or religious restrictions on eating poultry [41]. In accordance with The World Cancer Research, eating a lot of red meat (more than 500 g per week) can be harmful to health [42]. Poultry meat remains the simplest, quickest, and most cost-effective way to obtain high-quality animal protein. Chicken is high in protein and a good source of all necessary amino acids, with a lower saturated fat content than beef fat and no carbohydrates, making it an excellent choice for people trying to reduce weight or who suffer from conditions like cardiovascular disease. In addition, According to the Collaborative Research Support Program in Nutrition, the largely plant-based diets of children in rural areas of Egypt, Kenya, and Mexico were found to be significantly lower in micronutrients: vitamin A, vitamin B12, riboflavin, calcium, iron, and zinc. Chicken meat is a very rich source of all these elements, and when added to the diet, it can greatly enhance the nutritional content [43]. However, chicken and other meats have limited shelf stability [1,2]. The purpose of this study was to evaluate the effect of Moringa and olive leaves, and their extracts, on fat oxidation in chicken burgers under refrigerated storage, and to study the physical, chemical, and sensory attributes of chicken burgers. Four different treatments of Moringa and olive leaves and their extracts (powder and methanolic extract) were compared. This is the first study to evaluate the effects of Moringa and olive leaf preparations on chicken burger quality. The findings of this study will improve our understanding of using Moringa and olive leaves in the preservation of chicken burgers, and other processed meat products.

## 2. Materials and Methods

### 2.1. Materials

Methanol, sodium nitrite (NaNO_2_), BHT, aluminium chloride (AlCl_3_), sodium hydroxide (NaOH), Folin-Ciocalteu reagent, 2,2-diphenyl-1-picryhydrazyl radical (DPPH), gallic acid, sodium carbonate (Na_2_CO_3_), distilled water, magnesium oxide, boric acid, Tashiro indicator, HCl, filter paper, antifoaming agent, chloroform, acetic acid, potassium iodide, starch, and sodium thiosulfate (Na_2_S_2_O_3_.5H_2_O) were purchased from Sigma-Aldrich (Riyadh, Saudi Arabia). Moringa (*oleifera*) leaves were obtained from a local market in Al-Ahsa, Saudi Arabia. Olive leaves (*Olea europaea* L. *var. koroneiki*) were collected after harvesting fruits in October 2020, from olive tree farms in Al-Jouf, northwest Saudi Arabia.

### 2.2. Sample Preparation

Samples of fresh Moringa and olive leaves were cleaned by hand, washed with distilled water to remove dust and foreign contaminants, and dried at (35–40 °C). The dried leaves were crushed using an electric blender, sieved through a fine mesh, and stored in polyethylene bags at 4 °C, according to the method described by Singh and Immanuel [44].

### 2.3. Methanol Extraction

An orbital shaker (from Light Duty Orbital Shakers, Parsippany, NJ, USA) was used for extraction preparation (10 min, 100 rpm). Dry powdered Moringa and olive leaves (100 g) were individually mixed with 1500 mL of 80% methanol at 30 °C for 5 h at 500 rpm. Extracts were filtered using a Whatman #41 filter paper. The methanolic extracts were evaporated under reduced pressure for 3 h at 40 °C, using a rotary evaporator. The methanolic extract obtained was placed in an oven for drying for 24 h at 50 °C to obtain the dried, residue extracts [44].

### 2.4. Extraction Yield

After evaporation, the extracts were concentrated by oven drying and weighed to determine the extraction yield, which was calculated following Mahmoud et al. [45].

$$\mathrm{Extraction}\;\mathrm{yield}\;\left(\%\right)\;\;=\;\frac{\mathrm{Weight}\;\mathrm{of}\;\mathrm{the}\;\mathrm{extract}}{\mathrm{Total}\;\mathrm{weight}\;\mathrm{of}\;\mathrm{the}\;\mathrm{Row}\;\mathrm{material}}\;\times \;100$$

### 2.5. Total Phenolic Content (TPC)

The TPC was determined using the Folin–Ciocalteu technique [46]. Briefly, 2 g of each the dried Moringa, olive leaves, and their crude extracts were added to beakers, and 20 mL of methanol acidified with HCl (10%) was added, sonicated for 10 min in an ultrasonic bath, centrifuged for 10 min at 4000× *g*, and filtered. Subsequently, the sample (0.5 mL) was placed into test tubes to which 1.5 mL of Folin–Ciocalteu reagent was added, and the mixture was allowed to rest for 5 min at 25 °C. After another 5 min, 2 mL of NaCO_3_ (7%) was added, and the sample was incubated in the dark for 45 min at 25 °C. After incubation, the mixture turned blue, and 10 mL of distilled water was added to dilute the solution. The absorbance of the blue solution in various samples was determined at 715 nm, using a UV-18000 spectrophotometer (Shimadzu, China). The total phenolic content was calculated as mean ± SD (*n* = 3) and expressed as mg/100 g of gallic acid equivalent (GAE) of the dried samples.

### 2.6. Total Flavonoid Content (TFC)

A colorimetric assay was used to determine the presence of flavonoids [47]. Briefly, 4 mL of water was added to 1 mL of the methanolic of dried Moringa, olive leaves, and their crude extracts, separately. Subsequently, 0.3 mL of NaNO_2_ (50 g/L, *w*/*v*) and 0.3 mL of AlCl_3_ solutions (100 g/L) were added. After 5 min of incubation at room temperature, 2 mL of 1 M NaOH was added. The reaction mixture was immediately added to distilled water, to a final volume of 10 mL. The solution was vortexed, and the absorbance of the pink solution was measured at 510 nm using a UV-18000 spectrophotometer (Shimadzu, China). The TFC was calculated using the rutin calibration, and the results were presented as mg/100 of rutin equivalent (RE) of the dried sample (mg/100 g).

### 2.7. DPPH Radical-Scavenging Assay

Antioxidant activity was determined using the DPPH assay [48]. Briefly, 1 mL of each methanolic solution of the Moringa, olive leaves, and their crude extracts at various concentrations (40, 80, 120, 160, and 200 mg/mL) was added to 3 mL of DPPH in methanol (0.33%). The absorbance at 517 nm was measured using a UV spectrophotometer after 30 min at 37 °C. In this assay, methanol served as a blank control, and all extracts and controls were examined in triplicate. The scavenging effect (%) was calculated using the following formula:

$$\mathrm{Scavenging}\;\mathrm{Effect}\;\left(\%\right)\;\;=\;\frac{(\mathrm{Control}\;\mathrm{absorbance}\;-\;\mathrm{Test}\;\mathrm{absorbance})}{\mathrm{Control}\;\mathrm{absorbance}}\;\times \;100$$

The scavenging activity of DPPH was plotted against the concentration, and the IC_50_ value (the extract concentration that scavenges 50% of the radicals) was calculated via linear regression.

### 2.8. Chicken Burger Preparation

The chicken burgers were prepared using chicken meat purchased from local markets. They comprised 10% breast meat, 55% thigh meat, and 15% fat from the chicken skin, according to Fishler [49]. The chicken meat was minced and mixed with spices and cool water (13.8%). The completely homogenised chicken meat was divided into eight samples:

I. Control sample (C): chicken burgers without any treatment
II. Chicken burger + 0.01% BHT (BHT)
III. Chicken burger + 1% Moringa leaf powder (MLP1%)
IV. Chicken burger + 2% Moringa leaf powder (MLP2%)
V. Chicken burger + 0.02% Moringa leaf extract (MLE)
VI. Chicken burger + 1% olive leaf powder (OLP1%)
VII. Chicken burger + 2% olive leaf powder (OLP2%)
VIII. Chicken burger + 0.02% olive leaf extract (OLE)

Each chicken burger weighed 100 g and was shaped by hand using a mold plate (112 mm diameter × 2 cm height, HP 112; Picelli, Rio Claro, Brazil). Subsequently, the burgers were individually wrapped in polyethylene plastic and stored in a refrigerator at 4 ± 1 °C. All samples were stored for 20 days, and oxidation measurements were performed on Day 0 (at production), Day 10, and Day 20 (end of storage).

### 2.9. Total Volatile Nitrogen (TVN)

To measure the TVN, samples (10 g) were added to 100 mL distilled water and rinsed into a distillation flask containing 100 mL distilled water, and then 2 g of magnesium oxide and an antifoaming agent were added. A micro-Kjeldahl distillation device (Kjeltec system 2020 digestor) was used to dissolve the mixture. In total, 25 min of distillation was performed in 25 mL boric acid (4%), containing 5 drops of Tashiro indicator. To calculate the TVN in the sample, in terms of mg TVN/100 g, the solution was titrated with 0.1 M HCl (Pearson, 1976). The TVN concentration was calculated using the following formula [50]:

$$\mathrm{TVN}\;(\mathrm{expressed}\;\mathrm{in}\;\mathrm{mg}/100\;g\;\mathrm{sample})\;\;=\;\frac{((\mathrm{Vi}-V0)\;\times \;0.14\;\times \;2\;\times \;100)}{M}$$

Vi = Volume of 0.01 M hydrochloric acid solution in mL for sample; V0 = Volume of 0.01 M hydrochloric acid solution in mL for blank; M = Weight of sample in g.

### 2.10. Peroxide Value (PV)

The PV was calculated using the following technique (A.O.A.C.2000, Number 965.33), with certain modifications. First, 15 mL of hexane was added following centrifugation at 10,000 rpm for 10 min, which was performed twice to obtain de-fatted chicken burgers. Hexane was removed by heating, and the recovered oil was used to calculate the PV. A 2 g sample of oil was dissolved in 20 mL chloroform: acetic acid (1:2, *v*/*v*), and then 1 g of potassium iodide was added following heating in a water bath for 1 min to extract the oil. Subsequently, 20 mL potassium iodide solution (5%) and 50 mL of distilled water were added. In the presence of 0.5 mL of starch solution as an indicator, the iodine released was titrated with sodium thiosulfate (0.1 N). PV was measured in milliequivalents per kilogram, and was calculated using the following equation:

$$\mathrm{PV}\;\left(\mathrm{meq}/\mathrm{kg}\right)\;=\;\frac{(S\;-\;B)\;\times \;N\;\times \;1000}{G}$$

S = mL Na_2_S_2_O_3_ of the sample; B = mL Na_2_S_2_O_3_ of blank; N = normality of Na_2_S_2_O_3_; G = sample weight.

### 2.11. pH Value

The pH was determined by homogenising 10 g of material in 50 mL distilled water, after which the pH was measured using a digital pH meter (Benchtop pH Meters Hanna Instruments, Italy) [51].

### 2.12. Drip Loss

The difference between the weight of a completely frozen and an identical burger after thawing was used to calculate drip loss. Drip loss was calculated as a percentage of weight change [52].

### 2.13. Cooking Loss

The cooking loss of the prepared chicken burgers was calculated using the following method: [53]

$$\mathrm{Cooking}\;\mathrm{loss}\;\left(\%\right)\;=\;\frac{\mathrm{Raw}\;\mathrm{sample}\;\mathrm{weight}\;-\;\mathrm{cooked}\;\mathrm{sample}\;\mathrm{weight}}{\mathrm{Raw}\;\mathrm{sample}\;\mathrm{weight}}\;\times \;100$$

### 2.14. Cooking Yield

The cooking yield (%) was determined as specified by Zargar et al. (2014) [54]. The weight of each chicken burger was recorded before and after cooking. The following formula was used to calculate the cooking yield, which was then represented as a percentage:

$$\mathrm{Cooking}\;\mathrm{yield}\;\left(\%\right)\;=\;\frac{\mathrm{Weight}\;\mathrm{of}\;\mathrm{cooked}\;\mathrm{burger}}{\mathrm{Weight}\;\mathrm{of}\;\mathrm{raw}\;\mathrm{burger}}\;\times \;100$$

### 2.15. Sensory Evaluation

Chicken burgers were prepared based on standard specifications for burgers prepared in the Kingdom of Saudi Arabia [49]. Color, odor, flavor, texture, and general acceptability were evaluated by ten panellists on a ten-point scale as follows: excellent = 9–10, very good = 7–8, good = 5–6, acceptable = 3–4, and barely acceptable = 1–2, following Mahdi et al. (2016) [55].

### 2.16. Statistical Analysis

All measurements were performed thrice, and the results were presented as the mean standard deviation of triplicated experiments. The statistical analyses were carried out using the Statistical Package for the Social Sciences (SPSS) software, version 26 (IBM SPSS statistics, USA). Data were analyzed using ANOVA for all samples, and the results were compared using Duncan’s Test at a 5% significance level (*p* < 0.05).

## 3. Results and Discussion

### 3.1. Extraction Yield

The maximum yield of extracts from olive and Moringa leaves, using methanol as a solvent, was determined. The olive leaves showed a maximum yield of 24.65%, compared to the Moringa leaves, which showed a smaller extraction yield of 20.94%. The results obtained for the olive leaves were similar to those reported in Cho et al. (2020) [56], where the extract was obtained using 90% methanol, and the yield obtained was 20.41%. The results for the Moringa leaves were similar to those reported by do Nascimento et al. (2017) [57], who used 95% ethanol to obtain 25.022% (Table 1).

### 3.2. TPC

The phenolic content of the olive leaves was significantly (*p* < 0.05) higher (197.99 ± 9.199 mg GAE/100 g) than that of the Moringa leaves (162.98 ± 1.447 mg GAE/100 g). Moreover, crude olive extracts (926.18 ± 19.6 mg GAE/100 g) were found to have higher TPC than crude Moringa extracts (689.64 ± 83.1 mg GAE/100 g) (Table 2). Expectedly, the order of the TPC in the samples was: olive crude extract > Moringa crude extract > olive leaves > Moringa leaves. Similarly, Ghnimi et al. (2017) [9] reported high TPC in olive leaves (253 mg GAE/g). The TPC of Moringa leaves determined by the present study was similar to that reported by do Nascimento et al. (2017) [57]; in their study, the active phenolic compound concertation reported was 170.07 ± 0.43 mg GAE/g. In contrast, another previous study by Al-Owaisi et al. (2014) showed higher TPC (94.56 ± 3.53 mg GAE/g) in the crude Moringa (*M.* *peregrina*) extracts [58].

Several studies have reported the presence of polyphenols (tannins and flavonoids), steroids, alkaloids, carbohydrate glycosides, cardiac glycosides, and terpenoids in ethanolic extracts of Moringa and olive leaves. Studies on phenolic compounds have shown that they have potential biological properties such as antioxidant, antidiabetic, hepatoprotective, anti-inflammatory, antibacterial, and anticancer actions [59]. The biochemical and pharmacological characteristics of the phenolic compounds present in these medicinal plants are of great interest, mainly because of their anticarcinogenic and antioxidant actions [60].

### 3.3. TFC

The TFCs in Moringa, olive leaves, and their crude extracts were determined (Table 2). Overall, the flavonoid content was significantly (*p* < 0.05) higher in olive leaves (35.47 ± 0.55 mg/RE/100 g) and its crude extracts (208.82 ± 0.62 mg/RE/100 g) than in Moringa leaves (26.24 ± 0.22 mg/RE/100 g) and its crude extracts (14,767 ± 3.19 mg/RE/100 g). Our study results were similar to those of Unuigbe et al. (2015) [61], who evaluated the TFC in Moringa leaves using a methanol solvent, and reported a flavonoid content of 31.73 ± 2.66 mg/RE/100 g [61]. Another study by Xu et al. (2019) reported a higher flavonoid content (192.36 ± 2.96 mg/RE/g) for Moringa oleifera leaves crude extracts, compared to the values obtained in this study [62]. TFCs in crude olive extracts ranged between 42 and 57 mg/g in the study by Khaliq et al. (2015) [63], which was higher than that found in this study, which may be due to regional differences in olive leaves. Plant secondary metabolites such as flavonoids and tannins present in Moringa leaves are considered as promising polyphenolic compounds [64]. In the present study, the flavonoid content of olive leaves was similar to that determined by Abaza et al. (2011) [65] (21.47 ± 2.56 mg/RE/100 g).

### 3.4. DPPH Radical-Scavenging Assay

Free radical chain reactions are the most important process of lipid oxidation [10]. Antioxidants delay the oxidation of fats, thereby extending the shelf-life of food products [62]. Reactive oxygen species (ROS) are absorbed by antioxidants, which prevent them from damaging fat chains. Carotenoids, phenolics, tocopherol, and ascorbic acid are antioxidant molecules that neutralise ROS through free radical scavenging. In this study, the antioxidant properties of Moringa, olive leaves, and their crude extracts were determined using 2,2-diphenyl-1-picrylhydrazyle (DPPH), and expressed as IC_50_ values (the effective level of extract that inhibits 50% of the initial DPPH concertation). A decreased antioxidant activity of the plant extract was indicated by a greater IC_50_ value. Interestingly, the Moringa, olive leaves, and their crude extracts had IC_50_ values of 2.397 ± 0.10, 4.445 ± 0.01, 30.08 ± 0.02, and 36.88 ± 0.01 mg/mL, respectively (Table 2). The antioxidant activities of Moringa leaves were found to be the highest, whereas the antioxidant activities of the crude extract of olives were found to be the lowest. Some of these findings were similar to those of Xu et al. (2019), who reported an IC_50_ value for Moringa leaves of 1.87 ± 0.03 [62]. The results for crude olive extracts were consistent with the findings of Khaliq et al. (2015) [63]. The antioxidant activity of phenolic compounds is related to their structure, which enables them to function as metal chelators, and absorb and neutralise free radicals [66]. The degree of discoloration (reduction in absorbance) of the DPPH solution indicates the scavenging potential of the sample antioxidant during the free radical reaction. Plant secondary metabolites such as alkaloids, tannins, saponins, glycosides, and other metabolites are present in Moringa, olive leaves, and their crude extracts. Due to their hydrogen-donating capacity, all of these bioactive metabolites show antioxidant activities [64].

TVN is an indicator of poultry product quality, due to its association with certain microorganisms which remove the carboxylate group of amino acids [67]. Therefore, we evaluated the TVN content in chicken burger samples during refrigerated storage for 20 days (Table 3). An increase in TVN in chicken burgers was observed during the storage period, possibly due to the activity of microbes and their enzymes, which degrade proteins [68]. Significant differences (*p* < 0.05) in the TVN values were observed after storage for all samples. There were significant differences in the TVN content between the control and treated samples throughout the storage period. At day 10 of storage, chicken burgers treated with MLP2% showed the lowest TVN values, which was significant compared to that of other samples. At day 20 of storage, the MLE sample showed the lowest TVN value (*p* < 0.05) compared to the other samples. The MLE was more effective as a natural antioxidant and antimicrobial than the BHT during the late storage period. OLE treatment may reduce protein hydrolysis and TVN content. These results were similar to those reported in Saleh et al. [69]. The effects of MLP treatment were similar to the findings in Zeid et al. (2016) [70]. Plant extracts contain antibacterial compounds that can reduce the ability of bacteria to decrease the oxidative amination of non-protein nitrogen compounds [71]. OLEs function as an antioxidant and anti-microbial agent because of the presence of phenolic compounds that play a role in meat stabilisation during storage by reduction of bacterial activity [72,73].

### 3.5. PV

The amount of oxidation products increased significantly (*p* < 0.05) in the control and in all the chicken burger samples, as the storage duration increased from 0 to 20 days (Table 4). Between 0 to 10 d of storage, samples treated with Moringa and olive leaves, at concentrations of 1 and 2%, showed no significant differences in PV. There was no significant difference (*p* > 0.05) between the PVs of the BHT sample and the extract samples (MLE/OLE) throughout the storage period. BHT had the same effect on fat oxidation in chicken burgers. MLP contains polyphenols and flavonoids that can act as antioxidants, thus reducing fat oxidation and PVs [29,36]. MLP has a high capacity to prevent lipid oxidation in burgers by acting as a hydrogen donor, reducing agent, and singlet oxygen-scavenger [74]. Additionally, MLE has been reported to be highly effective against fat oxidation in meat products [50,75,76]. In the present study, the PV associated with olive leaves was similar to that reported by Albertos et al. (2018) [77]. OLEs contain different types of phenolic compounds, such as hydroxytyrosol and oleuropein, which act as antioxidants, and can delay the oxidation of fats, thus extending the shelf-life of food products [78].

### 3.6. pH Value

Significant differences (*p* < 0.05) were observed in the pH values of all samples throughout the storage period. The lowest pH values were observed in meat treated with the MLP and MLE. There was no significant difference in the pH values between the two groups (*p* > 0.05) (Table 5). Samples of chicken burgers treated with OLE and OLP1–2% showed significant differences in pH, compared to the control, throughout the storage period. The results of the olive leaf treatments agreed with those of Al-Rimawi et al. (2017) [78], whereas those of the Moringa leaves were similar to those of Zeid (2016) [70]. There was a slight decrease in the pH of samples treated with olive and Moringa leaves during the storage period, which may be attributed to the natural phenolic components in OLE. These findings were in agreement with Shalaby et al. (2018) [79]. Thus, these natural compounds may reduce the pH and create an environment which is unsuitable for microbial growth in meat [7].

### 3.7. Drip Loss

Drip loss (%) in all chicken burger treatments was determined (Table 6). The BHT sample showed the highest drip loss (0.77 ± 0.011%); however, this was not significant compared to that of the control. The addition of Moringa and olive leaves to chicken burgers resulted in a decrease in the drip loss. Treatment with the extracts also reduced the loss ratio compared to the control, and the difference was significant. The results for Moringa leaves and the control were similar to those reported in Zeid (2016) [70]. The difference in drip loss between burger samples treated with leaves and the control may be attributed to the presence of fibres. Studies have found that the addition of dietary fibre improves the cooking properties of beef burgers by increasing its ability to retain water and reducing drip loss [80,81].

### 3.8. Cooking Yield and Cooking Loss

The cooking yield (%) of the chicken burger samples was determined (Table 6). Compared to the treated chicken burgers, the control sample showed a significant (*p* > 0.05) reduction in cooking yield. Treatment with MLP2% resulted in the highest increase in cooking yield compared to the control, and all chicken burgers treated, and this change was significant (*p* < 0.05). There were significant differences (*p* < 0.05) in the cooking yield between the MLP1% and MLP2% samples. There were significant differences (*p* < 0.05) in the cooking yield between the OLP1% and OLP2% samples.

The cooking loss (%) of the different chicken burger samples was also determined (Table 6). The control showed a significant (*p* < 0.05) decrease in cooking loss compared to that of the different chicken burger samples. There were no significant differences (*p* > 0.05) in cooking loss between BHT and the extract-treated (MLE and OLE) samples. The MLP2% treatment showed the lowest cooking loss compared to the different chicken burger samples (*p* < 0.05). The reduction in size of the chicken burger during cooking was attributed to protein denaturation, moisture evaporation, and distillation of melted fat. Loss in size may range from approximately 5–25% of weight; however, fibre supplementation has the advantage of reducing moisture and weight loss [80,82,83].

### 3.9. Sensory Evaluation

The effects of Moringa and olive leaf treatments on the sensory parameters of the chicken burgers were determined (Table 7). Sensory factors represent an important indicator of potential consumer preferences based on organoleptic characteristics, such as color, flavor, texture, and general acceptability. MLP and OLP treatment did not negatively affect the color, as there was no significant difference (*p* > 0.05) between the color of the treatment and control samples. The flavor was affected to a certain extent by the addition of OLP2%, which had the lowest value compared to that of the other samples. The flavor of the burgers was not negatively affected by treatment compared to the control. Treatment of the chicken burgers, either with BHT or test samples, did not negatively affect the odor. There were no significant differences (*p* > 0.05) in the texture of the samples treated with MLP, OLP, MLE, and OLE compared to the control. Regarding general acceptability, chicken burgers treated with OLP2% were the least acceptable among the samples, whereas there was no significant difference (*p* > 0.05) in general acceptability of the other samples compared to the control. Similarly, the result of no significant difference in the sensory characteristics of chicken burgers treated with Moringa and its extracts at concentrations (0.5%, 1%, and 2%) has been reported by another study [84]. However, Saleh et al. reported that the addition of OLE at 0.25, 0.5, and 1% greatly improved the sensory qualities and overall acceptability [69].

## 4. Conclusions

The results of this study indicated that Moringa and olive leaves contain rich sources of natural antioxidants and safe bioactive compounds such as phenols. Phenols play an important role in reducing fat oxidation, inhibiting protein degradation, and increasing fat stability and the shelf-life of chicken burgers, which are a highly consumed product. The total phenolic content was as follows: crude olive extract > crude Moringa extract > olive leaf > Moringa leaf. The total flavonoid content was found to be significantly (*p* < 0.05) higher in the olive leaf and crude extracts than in the Moringa leaf and crude extracts. The antioxidant activities of Moringa leaves were found to be high compared with those of the crude extract of olive leaves, and Moringa leaf extracts were found to be more effective as a natural antioxidant and antimicrobial than the BHT, following prolonged refrigerated storage. Olive leaf extracts may reduce protein hydrolysis and total volatile nitrogen content. However, Moringa leaf and extracts did not influence the sensory attributes of the chicken burgers. In general, Moringa and olive leaf treatments slowed the deterioration of meat and may be effectively used as preservatives to extend the shelf-life of chicken burgers.

## Figures and Tables

**Table 1:** Yield extracts from *Moringa oleifera* and olive leaves, per 100 g.

Plant Leaves	Extraction Yield (%)
Moringa leaves	20.94 ± 0.11
Olive leaves	24.65 ± 0.09

Values are means ± SD of a triplicate sample (*n* = 3).

**Table 2:** The total phenolic and flavonoid content, and the antioxidant activity (IC_50_) of Moringa and olive leaves, and their crude extracts.

Sample Extracts	Total Phenolic (mg/GAE/100 g)	Total Flavonoid (mg/RE /100 g)	IC_50_ Value of DPPH (mg/mL)
Moringa (leaf)	161.98 ± 1.44 ^A^	26.24 ± 0.22 ^A^	2.397 ± 0.10 ^A^
Olive (leaf)	197.99 ± 9.19 ^A^	35.47 ± 0.55 ^B^	4.445 ± 0.01 ^B^
Moringa (crude extract)	689.64 ± 83.1 ^B^	147.67 ± 3.19 ^C^	30.08 ± 0.02 ^C^
Olive (crude extract)	926.18 ± 19.6 ^C^	208.82 ± 0.62 ^D^	36.88 ± 0.01 ^D^

Values are means ± SD of a triplicate sample (*n* = 3). Values with different superscript letters are statistically different (*p* < 0.05). Total phenolic content expressed as mg GAE/100 g) of dry leaves and crude extract. Total flavonoids content expressed as mg RE mg/100 g of dry leaves and crude extract.

**Table 3:** Total volatile nitrogen (TVN) of chicken burgers during storage for 20 days, at 4°C.

Total Volatile Nitrogen (mg/100 g)		Storage Period (Day)	
Samples	Day 0	Day 10	Day 20
C	1.81 ± 0.28 ^Aa^	21.28 ± 0.28 ^Fb^	26.88 ± 0.28 ^Hc^
BHT	1.96 ± 0.28 ^Aa^	15.68 ± 0.28 ^Db^	24.08 ± 0.28 ^Fc^
MLP 1%	1.96 ± 0.28 ^Aa^	7.84 ± 0.28 ^ABb^	18.48 ± 0.28 ^Cc^
MLP 2%	1.77 ± 0.42 ^Aa^	7.56 ± 0.28 ^Ab^	13.44 ± 0.28 ^Bc^
OLP 1%	1.92 ± 0.28 ^Aa^	18.48 ± 0.28 ^Eb^	21.28 ± 0.28 ^Ec^
OLP 2%	1.96 ± 0.28 ^Aa^	8.12 ± 0.28 ^Bb^	21.28 ± 0.28 ^Ec^
MLE	1.96 ± 0.28 ^Aa^	10.08 ± 0.28 ^Cb^	10.64 ± 0.28 ^Ac^
OLE	1.77 ± 0.42 ^Aa^	10.08 ± 0.28 ^Cb^	19.04 ± 0.28 ^Dc^

C: control; BHT: butylated hydroxytoluene; MLP 1, 2%: Moringa leaf powder 1 and 2%, respectively; MLE: Moringa leaf extract; OLP 1, 2%: olive leaf powder 1 and 2%, respectively; OLE: olive leaf extract; within the same column, values with superscript capital letters are statistically different (*p* < 0.05); and within the same row, values with superscript uncapitalized letters are statistically different (*p* < 0.05).

**Table 4:** The peroxide values of different chicken burgers, treated during refrigerated storage for 20 days, at 4 °C.

Peroxide Value (mq/kg)		Storage Period (Day)	
Samples	Day 0	Day 10	Day 20
C	5.00 ± 0.50 ^Aa^	20.500 ± 0.50 ^Cb^	25.00 ± 0.50 ^Cc^
BHT	5.00 ± 0.50 ^Aa^	10.500 ± 0.50 ^Bb^	10.50 ± 0.50 ^Bb^
MLP 1%	5.00 ± 0.50 ^Aa^	5.00 ± 0.50 ^Aa^	10.00 ± 0.50 ^Bb^
MLP 2%	5.00 ± 0.50 ^Aa^	5.00 ± 0.50 ^Aa^	10.00 ± 0.50 ^Bb^
OLP 1%	5.00 ± 0.50 ^Aa^	5.00 ± 0.50 ^Aa^	10.00 ± 0.50 ^Bb^
OLP 2%	4.50 ± 0.50 ^Aa^	4.50 ± 0.50 ^Aa^	10.00 ± 0.50 ^Bb^
MLE	5.00 ± 0.50 ^Aa^	10.00 ± 0.50 ^Bb^	10.00 ± 0.50 ^Bb^
OLE	5.00 ± 0.50 ^Aa^	10.00 ± 0.50 ^Bb^	10.00 ± 0.50 ^Bb^

C: control; BHT: butylated hydroxytoluene; MLP 1, 2%: Moringa leaf powder 1 and 2%, respectively; MLE: Moringa leaf extract; OLP 1, 2%: olive leaf powder 1 and 2%, respectively; OLE: olive leaf extract; within the same column, values with superscript capital letters, are statistically different (*p* < 0.05); and within the same row, values with superscript uncapitalized letters are statistically different (*p* < 0.05).

**Table 5:** The pH values of different chicken burger samples during storage for 20 days, at 4°C.

pH Value		Storage Period (Day)	
Samples	Day 0	Day 10	Day 20
C	5.97 ± 0.10 ^Aa^	5.99 ± 0.04 ^Eb^	6.93 ± 0.13 ^Cb^
BHT	5.79 ± 0.08 ^Aa^	5.91 ± 0.03 ^Eb^	6.16 ± 0.01 ^Cc^
MLP 1%	5.33 ± 0.03 ^Aa^	5.50 ± 0.01 ^ABb^	5.97 ± 0.12 ^Bc^
MLP 2%	5.27 ± 0.03 ^Aa^	5.47 ± 0.03 ^Ab^	5.65 ± 0.00 ^Ac^
OLP 1%	5.67 ± 0.05 ^Aa^	5.80 ± 0.01 ^Cb^	5.96 ± 0.01 ^Bc^
OLP 2%	5.65 ± 0.02 ^Aa^	5.77 ± 0.03 ^Cb^	5.86 ± 0.01 ^Bc^
MLE	5.38 ± 0.06 ^Aa^	5.54 ± 0.01 ^ABb^	5.60 ± 0.04 ^Ac^
OLE	5.76 ± 0.08 ^Aa^	5.87 ± 0.03 ^Ca^	5.96 ± 0.02 ^Bb^

C: control; BHT: butylated hydroxytoluene; MLP 1, 2%: Moringa leaf powder 1 and 2%, respectively; MLE: Moringa leaf extract; OLP 1, 2%: olive leaf powder 1 and 2%, respectively; OLE: olive leaf extract; within the same column, values with superscript capital letters, are statistically different (*p* < 0.05); and within the same row, values with superscript uncapitalized letters are statistically different (*p* < 0.05).

**Table 6:** Cooking yield, cooking loss, and drip loss percentage of chicken burger samples.

Samples	Cooking Yield (%)	Cooking Loss (%)	Drip Loss (%)
C	76.54 ± 0.52 ^A^	23.45 ± 0.52 ^G^	0.74 ± 0.02 ^E^
BHT	78.22 ± 0.07 ^BC^	21.78 ± 0.07 ^EF^	0.77 ± 0.01 ^E^
MLP 1%	87.32 ± 1.14 ^F^	12.67± 1.14 ^B^	0.43 ± 0.04 ^C^
MLP 2%	89.66 ± 0.56 ^G^	10.34 ± 0.56 ^A^	0.36 ± 0.04 ^B^
OLP 1%	83.06 ± 0.92 ^E^	16.94 ± 0.92 ^C^	0.46 ± 0.02 ^C^
OLP 2%	81.30 ± 0.89 ^D^	18.70 ± 0.89 ^D^	0.27 ± 0.06 ^A^
MLE	78.74 ± 0. 21 ^C^	21.26 ± 0.21 ^E^	0.43 ± 0.02 ^C^
OLE	77.30 ± 0.31 ^B^	22.69 ± 0.31 ^FG^	0.57 ± 0.01 ^D^

C: control, BHT: butylated hydroxytoluene, MLP 1, 2%: Moringa leaves powder 1 and 2%, respectively. MLE: Moringa leaves extract, OLP 1, 2%: Olive leaves powder 1 and 2%, respectively. OLE: Olive leaves extract. Within the same column, values with different superscript capital letters are statistically different (*p* < 0.05).

**Table 7:** Sensory evaluation of chicken burger treated with moringa, olive leaves extracts.

Treatment	Color	Flavor	Odor	Texture	Overall Acceptable
C	8.50 ± 2.01 ^BC^	7.70 ± 2.62 ^BC^	7.90 ± 2.37 ^A^	8.00 ± 2.44 ^AB^	7.60 ± 1.95 ^BC^
BHT	9.30 ± 0.94 ^C^	9.00 ± 1.05 ^C^	8.20 ± 1.61 ^A^	9.80 ± 0.42 ^B^	9.40 ± 0.69 ^C^
MLP 1%	8.70 ± 0.94 ^BC^	7.90 ± 2.33 ^BC^	8.20 ± 1.54 ^A^	7.30 ± 1.56 ^A^	8.50 ± 0.85 ^BC^
MLP 2%	8.50 ± 2.17 ^BC^	8.60 ± 1.89 ^BC^	7.60 ± 2.41 ^A^	7.80 ± 1.75 ^A^	8.00 ± 1.76 ^BC^
OLP 1%	6.30 ± 3.56 ^AB^	6.30 ± 3.23 ^AB^	7.60 ± 3.06 ^A^	6.90 ± 2.37 ^A^	6.20 ± 3.70 ^AB^
OLP 2%	6.80 ± 2.65 ^AB^	5.00 ± 4.05 ^A^	8.20 ± 2.78 ^A^	7.20 ± 2.25 ^A^	5.20 ± 3.88 ^A^
MLE	8.60 ± 2.31 ^BC^	9.40 ± 0.69 ^C^	9.10 ± 1.19 ^A^	8.70 ± 1.70 ^AB^	9.20 ± 0.91 ^C^
OLE	5.90 ± 3.66 ^A^	8.11 ± 1.96 ^BC^	7.80 ± 3.15 ^A^	8.20 ± 2.39 ^AB^	7.60 ± 3.13 ^BC^

C: control, BHT: butylated hydroxytoluene, MLP 1, 2%: Moringa leaves powder 1 and 2%, respectively. MLE: Moringa leaves extract, OLP 1, 2%: Olive leaves powder 1 and 2%, respectively, OLE: Olive leaves extract. Within the same column, values with different superscript capital letters are statistically different (*p* < 0.05).

## Data Availability

Data is contained within the article.
